# FLAGS, frequently mutated genes in public exomes

**DOI:** 10.1186/s12920-014-0064-y

**Published:** 2014-12-03

**Authors:** Casper Shyr, Maja Tarailo-Graovac, Michael Gottlieb, Jessica JY Lee, Clara van Karnebeek, Wyeth W Wasserman

**Affiliations:** 1 Centre for Molecular Medicine and Therapeutics, Child and Family Research Institute, Vancouver, BC Canada; 2 Department of Medical Genetics, University of British Columbia, Vancouver, BC Canada; 3 Treatable Intellectual Disability Endeavour in British Columbia, Vancouver, Canada; 4 Bioinformatics Graduate Program, University of British Columbia, Vancouver, BC Canada; 5 Genome Science and Technology Graduate Program, University of British Columbia, Vancouver, BC Canada; 6 Division of Biochemical Diseases, BC Children’s Hospital, Vancouver, BC Canada; 7 Department of Pediatrics, University of British Columbia, Vancouver, BC Canada

**Keywords:** Rare Variant, Exome Sequencing, Whole Exome Sequencing, Open Reading Frame Length, Exome Variant Server

## Abstract

**Background:**

Dramatic improvements in DNA-sequencing technologies and computational analyses have led to wide use of whole exome sequencing (WES) to identify the genetic basis of Mendelian disorders. More than 180 novel rare-disease-causing genes with Mendelian inheritance patterns have been discovered through sequencing the exomes of just a few unrelated individuals or family members. As rare/novel genetic variants continue to be uncovered, there is a major challenge in distinguishing true pathogenic variants from rare benign mutations.

**Methods:**

We used publicly available exome cohorts, together with the dbSNP database, to derive a list of genes (n = 100) that most frequently exhibit rare (<1%) non-synonymous/splice-site variants in general populations. We termed these genes FLAGS for FrequentLy mutAted GeneS and analyzed their properties.

**Results:**

Analysis of FLAGS revealed that these genes have significantly longer protein coding sequences, a greater number of paralogs and display less evolutionarily selective pressure than expected. FLAGS are more frequently reported in PubMed clinical literature and more frequently associated with diseased phenotypes compared to the set of human protein-coding genes. We demonstrated an overlap between FLAGS and the rare-disease causing genes recently discovered through WES studies (n = 10) and the need for replication studies and rigorous statistical and biological analyses when associating FLAGS to rare disease. Finally, we showed how FLAGS are applied in disease-causing variant prioritization approach on exome data from a family affected by an unknown rare genetic disorder.

**Conclusions:**

We showed that some genes are frequently affected by rare, likely functional variants in general population, and are frequently observed in WES studies analyzing diverse rare phenotypes. We found that the rate at which genes accumulate rare mutations is beneficial information for prioritizing candidates. We provided a ranking system based on the mutation accumulation rates for prioritizing exome-captured human genes, and propose that clinical reports associating any disease/phenotype to FLAGS be evaluated with extra caution.

**Electronic supplementary material:**

The online version of this article (doi:10.1186/s12920-014-0064-y) contains supplementary material, which is available to authorized users.

## Background

Uncovering the genetic basis of human disease improves care for affected patients and their families by providing a diagnosis, refining genetic counseling, informing clinical management (incl. decision making on appropriate preventive measures and available treatments), and ultimately facilitation of unrelated affected families as well identification of novel targets for treatment [1]-[3]. Rare Mendelian diseases are caused by altered function of single genes and individually have a low prevalence (fewer than 200,000 people in the United States, or fewer than 1 in 2,000 people in Europe) [4] but collectively these affect millions of individuals worldwide [5]-[7]. The current best estimate on the number of rare genetic disorders is between 6,000 to 7,000 [7] based on the catalogue Online Mendelian Inheritance in Man (OMIM) [8], and a comprehensive reference portal for rare diseases (Orphanet) [9]; however, taking into consideration that the human phenome is far from fully characterized [10] together with higher estimates on rare-disease-causing genes based on human mutation rate and the number of essential genes [11], the number of rare genetic disorders is likely higher.

Next-generation sequencing (NGS) high-throughput technologies have revolutionized the discovery of gene defects causing rare human diseases by detecting genetic variations at base-pair resolution within an individual [12]-[14]. NGS is widely used to sequence either a portion of the human genome (~1%) by capturing the protein-coding sequences (known as whole exome sequencing, WES), or to sequence the entire human genome (known as whole genome sequencing, WGS). In particular, WES technology had been widely used to identify genetic basis of Mendelian disorders by sequencing the exomes of just a few unrelated individuals or family members, and has led to discovery of more than 180 novel rare-disease-causing genes with Mendelian inheritance patterns, according to the review published in November 2013 [7],[15] (the number continues to increase with some rapidity). Considering the estimates that genetic basis has been determined for about ~3,500 of the rare diseases [7], there remain thousands of rare-disease-causing genes to be uncovered.

With the increasing rate of the discovery of rare genetic variants, WES has the potential to identify the majority of the remaining rare-disease-causing genes in the near future. A major challenge in identification of the true pathogenic variants lies in the differentiation between a large number of non-pathogenic functional variants and disease-causing sequence variants in a studied family (in this study, the term “functional variant” is restricted to missense/nonsense and splice site variants). Current WES analyses of rare genetic disorders use similar approaches [16] to filter the observed variants to enrich for potential causal genes. Specifically, after the reads are mapped, and variants are called and annotated, the variants are compared against internal exome databases as well as public databases, such as dbSNP [17], Exome Variant Server (EVS), 1000 Genomes Project [18], and HapMap project [19],[20] to exclude variants that are likely to arise from technological causes and variants that are common (e.g. variants observed in more than 1%) in a population. The variants are further prioritized based on their predicted effect on protein function [21],[22], where silent and non-coding variants (except for splice-site affecting variants) are typically excluded or ranked lower. The still extensive lists of candidate disease-causing variants can be further refined based on the family history and a hypothesized model of inheritance [7],[15]. However, it is well-established that a significant proportion of coding variants in each individual represent rare variants (absent from dbSNP or observed with frequency of ≤1%) [17],[20], and that genomes of healthy individuals contain an average of ~100 loss-of-function variants [23]. The analyst must further consider the possibility that non-coding variations (e.g. regulatory alterations) could be involved, thus the filtered results may not contain the causal gene. Thus, for many rare disorders, it is still challenging to separate the real disease-causing variant from the prioritized set of rare, likely functional variants that are not accountable for the investigated phenotype.

There are broadly used tools such as SIFT [21] and PolyPhen-2 [24] that provide an interpretation of mutation impacts. Many of these tools focus on the individual variants. In the variant-focused studies, it has been noted that variants tend to arise more frequently in long genes (e.g. *TTN* and *MUC16*). In considering that researchers often focus their interpretation of exome data on the genic level initially, it might be advantageous to have methods and ranking systems that integrate the individual variants at the genic level more systematically to inform variant prioritization. While there are long-standing methods for ranking a set of genes based on their annotations [25], there has been limited work on rankings based on sequencing properties. One ranking system based on the genic level is RVIS [26]. RVIS generates a score based on the frequencies of observed common coding variants compared to the total number of observed variants in the same gene.

To further help in identification of disease-causing variants from families affected by rare Mendelian disorders, we expanded the current, common prioritization parameters that focus mainly on frequency at which variants themselves are seen in normal population, to include the frequency at which genes are found to be affected by rare, likely functional variants. Using rare variations from dbSNP and EVS, we introduced the concept of FLAGS (FLAGS for FrequentLy mutAted GeneS). We showed that these genes possess characteristics that make them less likely to be critical for disease development, but are more likely to be assigned causality for diseases than expected for protein-coding genes in general. We further demonstrated FLAGS’ utility via a case study as well as literature review, and application in our in-house database. Finally, we provided a ranking system from FLAGS to assist in the prioritization of genes from exome/whole-genome clinical studies.

## Methods

### Terminologies used in this study

In this study, the term “functional variants” refers to variants that are missense, nonsense or fall within a splice site window (see below for specifics). The length of a gene is defined to be the longest open reading frame (ORF) of the gene, thus excluding promoters, untranslated regions and introns. All genes are referred to by their HGNC (HUGO Gene Nomenclature Committee) [27] official gene symbol.

### Datasets

In the following sections, we provide detailed descriptions of how the datasets were obtained or generated. Table 1 lists the size and descriptive nature of the datasets used in this study. Each gene list referred to in this report can be found in Additional file 1: Table S1.

- **FrequentLy mutAted GeneS (FLAGS)**

Variations from EVS hosted on the NHLBI Exome Sequencing Project (ESP6500) were downloaded on February 2014. The criteria used to generate the variations are available online (http://evs.gs.washington.edu/EVS/). Variations from dbSNPv138 [17] were downloaded from the NCBI website (version date 20130806). Genomic annotations were assigned to each variation using SNPeff v3.5g [29] with the parameter –SpliceSiteSize 7 and human genome version GRCh37.75. Variants were filtered for allelic frequency <1% according to dbSNP’s overall frequency and EVS’s combined population frequency. Where a discrepancy in the reported frequency arose between the two resources, we took the higher frequency. Variants were further filtered for “functional” coding mutations that result in a change in the amino acid sequence (i.e. missense/nonsense), or mutations that reside within a putative splice site junction (with a window size of 7, as supplied in the parameter for SNPeff). The remaining mutations were excluded if they were observed more than 10 times within our in-house database consisting of 150 exomes and 13 whole genomes (a list of filtered out variants are provided in Additional file 2: Table S6 as VCF). This last step was included because we noticed it is common to see polymorphic mutations from dbSNPv138 without an allelic frequency attached; filtering against an in-house pipeline allowed us to remove polymorphic variants that do not have an annotated frequency. Among these remaining mutations, for each gene, we counted the number of mutations observed per gene. Only protein-coding genes with a fully annotated translation start and end, and a valid dN/dS ratio are included for consideration (see Methodology section “Gene length and dN/dS ratio”). From this ranked list, we selected the top 100 genes (0.5% of the 19818 genes overlapping between dbSNP and EVS) with the most observed mutations as a focus for this study. This set will be referred throughout the manuscript as “FLAGS”. The entire ranked list is available in Additional file 3: Table S4.

b. **Disease genes datasets**

To obtain a list of reliable disease-associated genes, we drew from multiple resources. The first list of disease-associated genes was downloaded from OMIM website on March 2014 using the provided file “morbidmap”. This list will be referred throughout the manuscript as “OMIM genes”. A second list contains pathogenic variations downloaded from the HGMG professional version (file date 20130927) [28]. To focus on likely high-penetrance pathogenic alleles, we filtered the variations in this file by the same frequency criteria as we performed for obtaining FLAGS (see Methodology section “FrequentLy mutated GeneS”), and limited to only the mutations annotated as “DM” (damaging mutations). The affected genes from those remaining variations are compiled, and will be referred throughout this manuscript as “HGMD genes”. A third disease set was downloaded from the Supplemental file published by Boycott et al. (2013) [7], which provided a compiled list of novel genes and/or novel phenotypes associated with known disease-genes discovered through exome sequencing. For all three disease-associated-gene lists, we mapped the gene symbols to their official HGNC gene symbol (and discarded the ones that could not be mapped), retained only protein-coding genes with a fully annotated translation start and end, and a valid dN/dS ratio. OMIM and HGMD (Human Gene Mutation database) overlap with the top 100 FLAGS by 42 and 37 genes respectively (Additional file 4: Table S2A, S2B).

c. **Background dataset**

The complete list of human-coding genes was downloaded from Ensembl [30] Biomart on March 2014 using version Ensembl Genes 75 with genome version GRCh37.p13. Protein-coding genes without HGNC gene symbol, a proper translation start and translation end annotation according to this genome version were discarded. Genes without a valid dN/dS ratio were removed (i.e. without any observed synonymous polymorphisms according to dbSNPv138 and EVS). This last step was done for two reasons: 1) to ensure there is no bias when evaluating dN/dS ratio in our results, 2) to ensure the genes selected in this study have been covered in NGS studies, since any gene without at least one observed synonymous mutation is presumably not sufficiently captured in either exome or whole-genome studies. The Background set overlaps FLAGS completely.

The comparison analyses in the Results section are done without removing the overlap between the gene datasets.

**Table 1:** **Description of the datasets used in this study**

Name of datasets	Size	Description
FLAGS	100	The top 100 of FrequentLy mutAted GeneS with rare (<1% allelic frequency) functional variants from dbSNPv138 and ESP6500
OMIM	3099	The list of protein-coding genes associated with human diseases from Online Mendelian Inheritance in Man [8]
HGMD	2691	The list of protein-coding genes with damaging mutations (<1% allelic frequency) from Human Gene Mutation Database [28].
WES	300	Downloaded from Boycott et al. (2013) [7] - a list of novel genes implicated in human disorders based on whole exome sequencing studies, or novel/known pathogenic mutations discovered by whole-exome sequencing.
Background	18580	The entire set of human protein-coding genes that have complete start and end translation annotations with a specified dN/dS ratio

### Gene length and dN/dS ratio

We calculated the selection pressures acting on genes by comparing non-synonymous substitution per non-synonymous site (dN) to the synonymous substitutions per synonymous site (dS). This ratio of the number of non-synonymous substitutions per non-synonymous site to the number of synonymous substitutions per synonymous site (dN/dS) was calculated using the formula $\frac{\frac{\#\;\mathrm{of}\;\mathrm{observed}\;non-\mathrm{synonymous}\;\mathrm{substitutions}}{\#\;\mathrm{of}\;\mathrm{possible}\;non-\mathrm{synonymous}\;\mathrm{site}}}{\frac{\#\;\mathrm{of}\;\mathrm{observed}\;\mathrm{synonymous}\;\mathrm{substitutions}}{\#\;\mathrm{of}\;\mathrm{possible}\;\mathrm{synonymous}\;\mathrm{substitutions}}}$ [31]. The number of possible synonymous and non-synonymous mutations was derived by examining the longest annotated coding transcript per gene (transcript length based upon Ensembl Biomart described above). Only transcripts with annotated start and end positions were considered. The number of observed synonymous and non-synonymous mutations was calculated from the same dbSNPv138 and EVS datasets as described above. We verified that our methodology provides a comparable dN/dS ratios to the ratios reported previously [31] (Additional file 5: Table S5). Gene length was derived by converting the same transcript that was used to calculate the dN/dS ratio into amino acid sequences. In this study, the term “gene length” is defined to be the ORF of the gene, thus excluding promoters, untranslated regions and introns.

### Paralogs

The paralogous relationships for human genes were derived from the Ensembl Comparative Genomics API using version Ensembl Genes 75, GRCh37.p13. A custom Perl script was written to extract the paralogs for every gene.

### Gene-to-disease phenotypic terms

We used MeSHOP software [32] to identify over-represented disease terms associated with each gene. MeSHOP returns a list of MeSH (Medical Subject Heading) terms for each gene with a p-value for each term. Each p-value was calculated by an over-representation (compared to control) of the MeSH terms assigned to the set of articles within PubMed that are associated with the gene (based on relationships defined in gene2pubmed; articles considered include up to March 2013). From this output, for each gene, the non-disease related MeSH terms were filtered out, and the remaining MeSH terms were selected for significance (using the Bonferroni correction and a significance threshold of 0.05). To derive gene-to-disease relationships with an independent source, we extracted phenotypic diseased terms per gene from Human Phenotype Ontology website [33] by downloading the file “genes_to_diseases.txt” (version April 2014).

### Publication record analysis

For our publication analysis on the relationship between a gene and its frequency of citation(s) within biomedical literature, we used Gene Reference into Function (GeneRIF), a manually curated list of experimentally validated gene functions available as part of NCBI’s EntrezGene database. Each entry in GeneRIF contains a short description of a gene function and a PubMed identifier for the publication documenting the evidence of the described function. Therefore, we were able to count the number of papers published on a gene’s functionality by counting the number of PubMed records associated to the gene. The following are the detailed steps of our publication calculation. First, two flat files necessary for our analysis were downloaded via FTP from NCBI Gene on April 2014: GeneRIF (available at ftp://ftp.ncbi.nih.gov/gene/GeneRIF/generifs_basic.gz) and EntrezGene entries for human (ftp://ftp.ncbi.nih.gov/gene/DATA/Homo_sapiens.gene_info.gz). Second, because GeneRIF refers to each gene by its EntrezGene ID, we mapped the gene symbol of all genes on our lists (FLAGS, OMIM, HGMD, Background) to EntrezGene ID using EntrezGene entries downloaded in the previous step. Third, for each gene of interest, we counted the number of PubMed IDs (PMIDs) associated with its EntrezGene ID in GeneRIF. Because GeneRIF does not guarantee one-to-one relationship between a GeneRIF entry and a PMID (http://www.ncbi.nlm.nih.gov/books/NBK3840/#genefaq.Why_does_the_number_of_GeneRIFs), we filtered out duplicates in the list of PMIDs linked to a gene. Last, to filter the PMIDs by their publication date, we collected the publication date of each PMID via queries into PubMed using the ESummary query provided within the Entrez Programming Utilities (E-utilities).

### Statistical analyses

Unless stated otherwise, all statistical analyses and plots were carried out in R [34] version 2.15.3. Non-parametric Mann–Whitney U one-tailed test was executed by wilcox.test function with parameter exact = TRUE. Violin plots were generated with Vioplot package. The input files to the analyses are available in Additional file 6: Table S9A and 9B.

### Mutation Detection using WES – a case study

A 3-year old female patient, born as an only child to non-consanguineous parents of Turkish descent after an uncomplicated pregnancy and delivery, presented with profound early-onset developmental delay, microcephaly, seizures, dysmorphic features, myopia, bone marrow dysplasia with lymphopenia, neutropenia, aplastic anemia and combined immunodeficiency (B and T cell) was enrolled into the TIDEX gene discovery project, approved by the Ethics Board of the Faculty of Medicine of the University of British Columbia (H12-00067).

Extensive clinical investigations were performed according to the TIDE diagnostic protocol [35] to determine the etiology of patient’s condition. These included: chromosome micro array analysis for copy number variants (CNVs) (Affymetrix Genome-Wide Human SNP Array 6.0); telomere length analysis; CT and MRI scans and comprehensive metabolic testing.

Genomic DNA was isolated from the peripheral blood of the patient as well as parents using standard techniques. Whole exome sequencing was performed for the index patient and her unaffected parents using the Ion AmpliSeq™ Exome Kit and Ion Proton™ System from Life Technologies (Next Generation Sequencing Services, UBC, Vancouver, Canada) at 120X coverage. An in-house designed bioinformatics pipeline (Additional file 7: Text S3) was used to align the reads to the human reference genome version hg19 and to identify and assess rare variants for their potential to disrupt protein function. The candidate variants were further confirmed using Sanger re-sequencing in all the family members. Primer sequences and PCR conditions are available on request. Deleteriousness of the candidate variants was assessed using Combined Annotation–Dependent Depletion (CADD) scores [36].

## Results

### FLAGS: genes frequently affected by rare, likely-functional variants in public exomes

**Figure 1:**
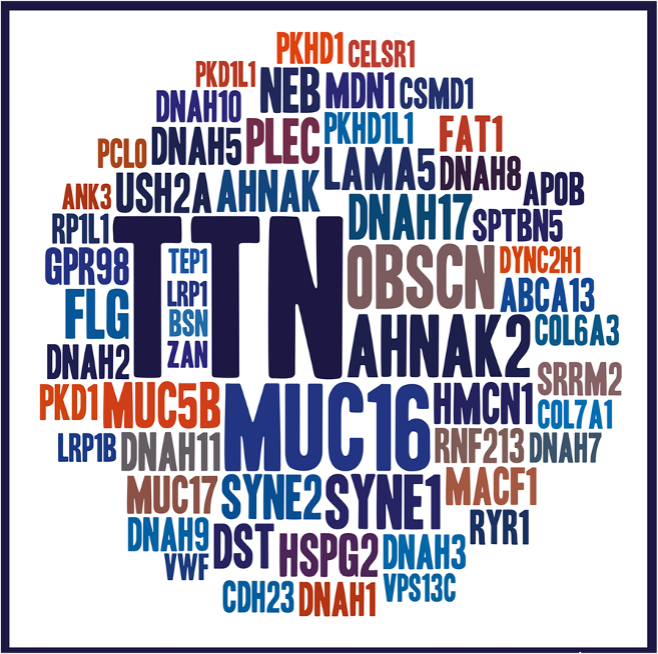
**The word cloud of FLAGS.** A text file was created using a custom Perl script to reflect the frequency of mutation per gene in FLAGS. The Tagxedo (http://www.tagxedo.com/) was then used to generate the word cloud. The size of the words reflects how frequently they are found to bear rare, likely functional variants in the general population. As expected *TTN* and *MUC16* are the top two genes.

It has been previously reported that *TTN* and *MUC16* appear in multiple exome analyses due to their length [37]-[41]; researchers are aware of these genes and are cautious when encountering rare likely functional (missense, nonsense, splice site) variants in WES analyses [37]-[41]. In a study of 53 independent families suffering from distinct rare inborn errors of metabolism (comprising of 150 whole exomes and 13 whole genomes; http://www.tidebc.org; Additional file 8: Text S4 and Additional file 9: Table S7), we confirmed that rare/novel, likely functional variants affecting *TTN* and *MUC16* repeatedly passed all the prioritization steps of our pipeline and appeared in ~5% of our candidate disease-gene lists. However, other genes were repeatedly observed in multiple families affected with different phenotypes (e.g. *DST*). This motivated us to compile a set of FLAGS (FrequentLy mutAted GeneS) to understand their properties and facilitate better interpretation of phenotypes associated with these variants. The FLAGS list was generated by ranking genes based on number of rare (<1%) functional variants affecting these genes in general populations (NHLBI Exome Sequencing Project (ESP6500) and dbSNPv138). As expected, *TTN* and *MUC16* are the top two genes based on the number of rare functional variants; however, other genes that were frequently affected by rare, likely functional variants in multiple TIDE families with unrelated phenotypes were also observed to be frequently mutated in general population (Additional file 10: Table S8). To explore the properties of these frequently mutated genes, we focused our analysis on the top 100 from this ranked list, which we hereafter refer to as FLAGS (Figure 1).

### FLAGS tend to have longer ORFs

**Figure 2:**
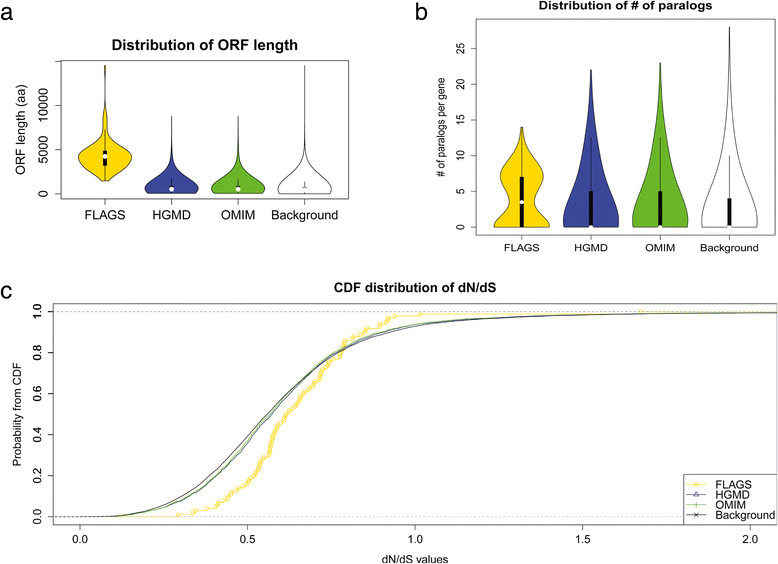
**Properties of FLAGS. (a)** Violin distribution of open reading frame lengths across the evaluated gene sets. Y-axis shows the length defined in terms of amino acids for the longest annotated transcript per gene. Outliers are excluded from the plot. **(b)** Distribution of number of paralogs per gene across the evaluated gene sets. Y-axis shows the violin distribution of paralogs based on Ensembl Compara database. Outliers are excluded from the plot. **(c)** Cumulative distribution of dN/dS ratio across the evaluated gene sets. X-axis is limited from 0 to 2, and Y-axis plots the corresponding probability according to the cumulative distribution function.

In this study, the assignment of gene length refers to the longest open reading frame. Genes with longer ORFs are expected to have more mutations than shorter genes. To confirm this, we determined the distribution of gene lengths based on the longest annotated open reading frame for each gene. FLAGS have an average length of 4653 ± 3605 aa (amino acids). The high variance is due to two genes (*TTN* and*MUC16*) having extremely long lengths (35992 and 14508 aa respectively) compared to the rest of the protein coding genes. Excluding the 2 outlying genes, the remaining FLAGS genes (n = 98) have an average ORF length of 4233 ± 1399 aa. Figure 2a shows the distribution of ORF lengths across different evaluated datasets (with outliers removed to show the distribution clearer). The entire FLAGS have overall much higher ORF length than HGMD, OMIM and Background (HGMD, OMIM comparisons each yield a p-value <2.2e^−16^, Background comparison yields a p-value of 0.00027). This is aligned with our expectation that FLAGS are frequently mutated from exome analysis because they correspond to genes with long coding regions.

### FLAGS tend to have paralogs

The presence of paralogs may increase tolerance for otherwise phenotype-inducing functional variations due to functional compensation [42],[43]. We calculated the number of paralogs per gene reported by the Ensembl Compara database [30], and compared this property between different gene sets. FLAGS overall have an average of 4 paralogs per gene. Figure 2b shows the distribution of the number of paralogs across the different gene sets. Aligned with our expectation, FLAGS have more paralogs than genes from OMIM, HGMD and Background (OMIM p-value = 7.2e^−05^, HGMD p-value = 7.4e^−05^, Background p-value = 8.1e^−09^). While the existence of paralogs may cause read mapping challenges that leads to an increased frequency of false variant predictions, most of these technical errors will be eliminated by a filter for variant frequency, as they will arise recurrently.

### FLAGS tend to have higher dN/dS ratios

Genes which exhibit many functional genetic variations (missense/nonsense/splice site) may have a higher tolerance for variations and thus a reduced likelihood of phenotypes subject to negative selection. For each gene, we calculated the dN/dS ratio as a proxy indicator of the amount of selective pressure acting on protein-coding genes. FLAGS have an average dN/dS ratio of 0.65 ± 0.18. Overall these genes have significantly higher ratio compared to genes from HGMD, OMIM, and Background (each individual comparison yields a p-value <0.005). Figure 2c shows the relative densities from cumulative distribution functions for each gene set. The trend indicates that frequently mutated genes have higher dN/dS ratio on average than expected.

### Variants detected in FLAGS tend to be predicted as less deleterious

**Figure 3:**
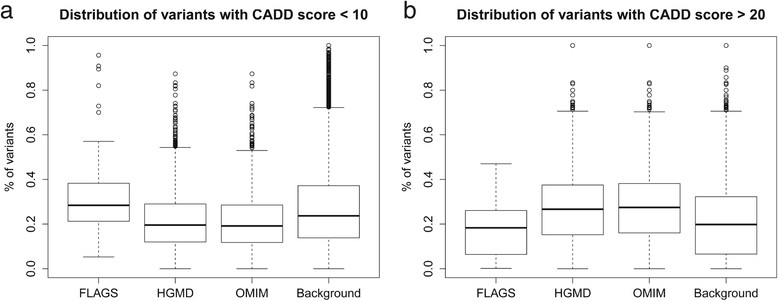
**FLAGS genes are affected by rare variants predicted to be less deleterious than the variants affecting known disease-genes. (a)** A boxplot distribution of proportion of variants with CADD score <10. The Y-axis plots the proportion of variants within each gene set having a Phred-scaled CADD score of <10. The proportion was calculated per individual gene. **(b)** A boxplot distribution of proportion of variants with CADD score >20. The Y-axis plots the proportion of variants within each gene set having a Phred-scaled CADD score of >20. The proportion was calculated per individual gene.

We explored the possibility that the FLAGS genes are affected by less deleterious rare variants compared to other genes. If the variants in FLAGS are less likely to be involved in diseases, then we would expect the variants to have lower predicted damage scores. To calculate this, we used the Phred-scaled Combined Annotation Dependent Depletion (CADD) score developed by Kircher et al. (2014) to rank the deleteriousness of each single nucleotide variant [36]. The method objectively integrates diverse annotations into a single measurement for each variant by training upon ~15 million genetic variants separating humans from chimpanzees against a simulated set of variants not exposed to selection. This method was chosen over other variant prediction tools because of its superior performance [36] and its ability to quantify the severity of a variant by a ranking system. This ranking system compares the candidate variant against other possible variants in the genome and assigns it a score based on this comparison; other variant prediction tools do not take into account other possible mutations in the genome [44]. Also, the CADD method includes ranking of nonsense and splice site variants, while other tools only handle missense [36]. For each gene, we calculated the proportion of variants with CADD Phred-scaled score <10, between 10 and 20, and above 20. We found that FLAGS are more enriched for variants with low scores, compared to OMIM and HGMD (Figure 3a; p-values = 2.6e^−11^, 2.9e^−12^ respectively). Likewise, OMIM and HGMD are more enriched for variants with high impact score (>20) than FLAGS (Figure 3b; p-values = 2.4e^−09^, and 1.2e^−10^ respectively). These results are aligned with our expectation. We additionally analyzed the genic tolerance of FLAGS to functional genetic variants, using residual variation intolerance score (RVIS) published by Petrovski et al. (2013) [26] and observed trends in the same direction (Additional file 11: Text S2).

### FLAGS tend to be reported in PubMed and associated with disease phenotypes

**Figure 4:**
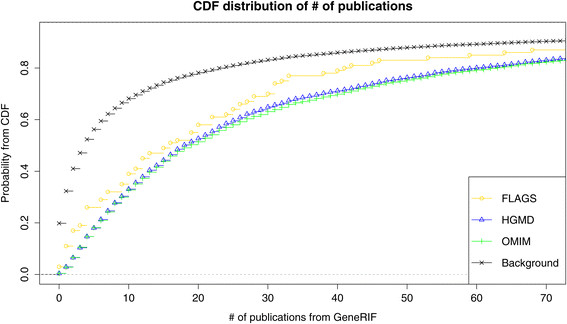
**Cumulative distribution of the number of publications per gene across the evaluated gene sets.** X-axis plots the number of publications from GeneRIF per gene, and Y-axis plots the corresponding probability according to the cumulative distribution function.

We sought to determine if there is a publication bias for pathogenic mutations in the frequently mutated genes. For each gene, we calculated the number of publications related to human diseases and biological functions using GeneRIF annotations (Figure 4). FLAGS have an average of 51 articles per gene, which is lower than for genes from HGMD and OMIM (OMIM p-value = 0.00087, HGMD p-value = 0.0035). However, FLAGS have more publications than the Background set (p-value = 6.3e^−12^).

**Figure 5:**
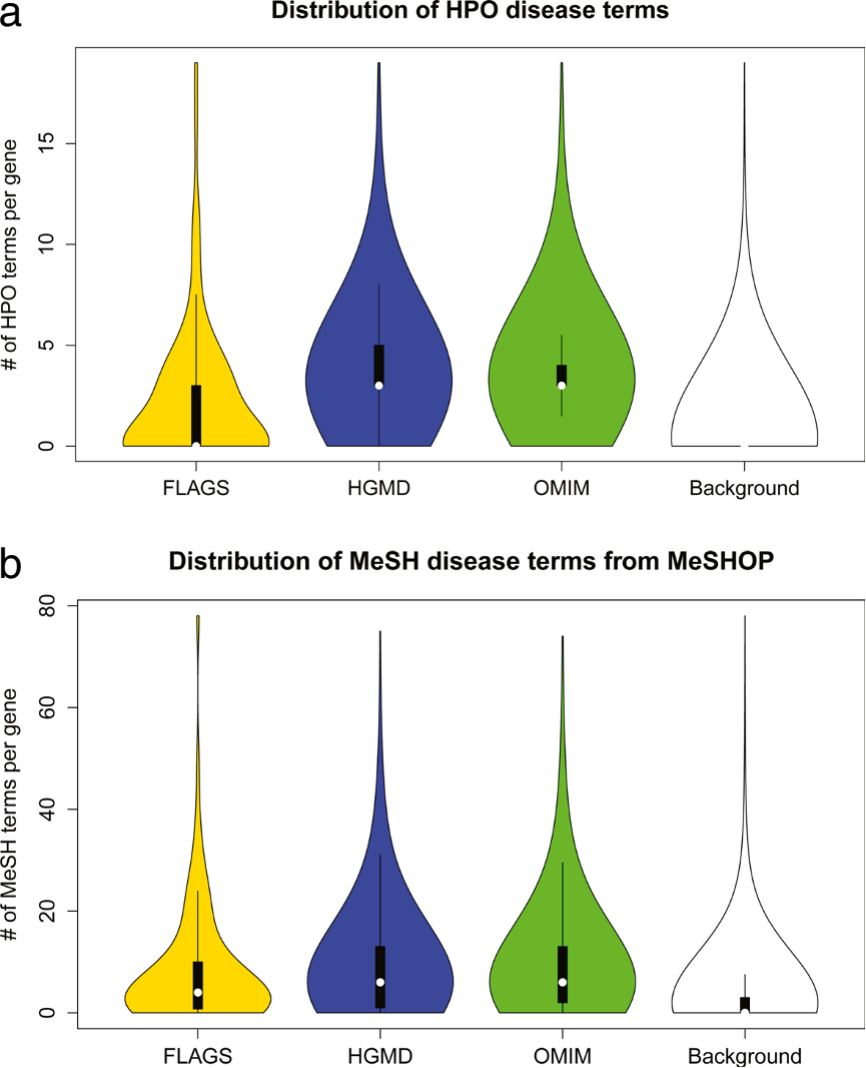
**FLAGS tend to be associated with disease phenotypes. (a)** Violin distribution of number of HPO disease terms across the evaluated gene sets. Y-axis is the violin distribution showing the number of HPO terms per gene. Outliers are excluded from the plot. **(b)** Violin distribution of number of MeSH disease terms from program MeSHOP across the evaluated gene sets. Y-axis is the violin distribution showing the number of MeSH terms per gene. Outliers are excluded from the plot.

We next considered if the frequently mutated genes are associated with greater diversity of disease phenotypes compared to disease-associated genes. Our expectation is that if the frequently seen genes are arising as candidates in more studies, and are less likely to be truly pathogenic, then they could be associated to a wider range of phenotypes in the literature (we recognize the association could also be due to pleiotropy [45], see Limitations). To analyze if FLAGS have been frequently correlated to human diseases, we used two different computational resources (MeSHOP [32], HPO [33]) to extract known significant relationship(s) between genes and human disease phenotypes based on published scientific articles. Figures 5a and b show the distribution of the number of disease terms from HPO and MeSHOP per gene within gene sets. From MeSHOP results, we see that FLAGS have slightly fewer MeSH diseased terms per gene than genes from OMIM (mean 8.1 vs. 10.2; p-value = 0.013), and significantly fewer terms per gene than HGMD genes (mean 8.1 vs. 9.5; p-value = 2.3e^−12^). FLAGS have more MeSH terms than Background genes (mean 8.1 vs. 3.1; p-value = 1.3e^−15^). These observations are consistent with the results based on HPO annotations, where we again see that while FLAGS have fewer disease phenotypic terms than genes from OMIM and HGMD (mean 2.1 vs. 3.7 and 3.8 respectively; p-values <0.0001), FLAGS exhibit more terms than the Background (mean 2.1 vs. 0.6; p-value = 3.7e^−14^). To adjust for the potential bias that genes with more articles are likely to have more MeSH and HPO terms attached, we repeated the analysis by normalizing the MeSH and HPO terms to the number of publications in GeneRIF. The normalized observations are consistent with the results if no normalization was applied (Additional file 12: Text S5).

### FLAGS recently implicated in rare-Mendelian disorders

We sought to determine which FLAGS have been reported with pathogenic mutations in NGS clinical studies. Boycott et al. (2013) provided a compilation of 178 novel genes discovered to be disease-associated through exome sequencing [7], of which three overlapped with FLAGS (*KMT2D/MLL2*, *HERC2*, and *DST*). To explore the properties of those 3 genes, we analyzed the ratio between number of rare variants and gene length, as well as presence of putative essential protein domains by assessing the distribution of rare variants across the gene. We found that among the FLAGS, *KMT2D* and *HERC2* have the lowest ratios of number of rare variants compared to gene length, while *DST* is one of the three genes among the FLAGS set with significant non-uniform distribution of rare variants across the gene (p-value = 1.2e^-04^; the other two are *EPPK1* and *HRNR*; see Additional file 13: Text S1 for more details on methodology and rationale). If we were to expand this 178 novel-rare-disease gene list from Boycott et al. (2013) to include the exome studies reporting on already-known disease-associated genes with known/novel pathogenic mutations, then this expanded set (n = 300) overlapped FLAGS by an additional 7 genes (*TTN*, *RYR1*, *PKHD1*, *RP1L1*, *ASPM*, *SACS*, *ABCA4*). In the discussion we provide our thoughts and literature analysis on why these genes have been reported as disease-associated despite being among the frequent genes to harbor rare functional variants.

### Applying FLAGS to prioritize candidate variants

#### Case study

To demonstrate a disease-causing variant prioritization approach using FLAGS and whole exome sequencing data, we selected one family from our TIDE cohort affected by an unknown rare genetic disorder. Through WES performed for the index and her unaffected parents (Methodology - Mutation Detection using WES – a case study), rare variants were identified and assessed for their potential to disrupt protein function. Only those variants predicted to be functional (missense, nonsense and frameshift changes, as well as in-frame deletions and splice-site effects) were subsequently screened under a series of inheritance models. In total, we identified six rare “functional” homozygous, and eight rare “functional” compound heterozygous candidates. Of those, only two genes affected by missense variants were considered functional candidates:

1. *VPS13B* gene (MIM 607817) had been found to bear homozygous or compound heterozygous mutations in patients with Cohen syndrome (MIM 216550). Cohen syndrome is characterized by developmental delay/intellectual disability, facial dysmorphism, microcephaly, neutropenia, and weak muscle tone (hypotonia). The features of Cohen syndrome vary widely in presence and severity among affected individuals. Additional features, perhaps patient-specific, appear in the reports; myopia and small hands and feet are observed in our patient. In our WES analysis, we identified two rare variants affecting this gene in the index, suggesting compound heterozygous inheritance. Neither of the variants was found in more than 160 in-house exomes; one of the variants was predicted to be deleterious using the CADD scores [36] with a score higher than 20, while the second variant was given the score of less than 5. Sanger re-sequencing confirmed that mother is a carrier of one variant, while the father is the carrier of the second variant and the index is compound heterozygous making the *VPS13B* gene a candidate disease-gene in this family.
2. *SENP1* gene (MIM 612157) product is one of the desumoylating enzymes [46] which is important for proper development and survival in mice. SENP1 was found to regulate expression of *GATA1* in mice and subsequent erythropoiesis [47]. Furthermore, SENP1 was found to be essential for the development of early T and B cells through regulation of *STAT5* activation [48]. To date, germline mutations in *SENP1* had not been described in any human diseases. Our WES analysis identified a rare missense homozygous variant in the index. The variant was not found in more than 160 in-house exomes and was predicted to be the most deleterious of all homozygous variants using the CADD scores [36]. The Sanger re-sequencing of the genomic DNA confirmed that index is homozygous for the variant, while both parents are carriers.

To further prioritize between these two genes, we consider a FLAGS-based approach. The *VPS13B* gene is one of the FLAGS (top 100, rank 67) and is frequently seen to be affected by rare, likely functional variants in general population. On the other hand, *SENP1* is rarely affected by functional variants in the general population (rank 11,947). In addition, *VPS13B* is a frequently seen in the TIDE cohort of patients, 22 of 160 individuals have rare, likely functional alleles in the *VPS13B* gene that pass our prioritization filters. In contrast, the family reported here is the only family from the TIDEX cohort of patients with a rare, likely functional variant affecting the *SENP1*. In none of the other 160 exomes did the variants in *SENP1* pass our prioritization filters for rare, likely functional variants. Together with the fact that *VPS13B* does not fit well to her severe hematologic findings and bone marrow dysplasia, FLAGS helped us select *SENP1* as candidate gene for our experimental validation studies. The case report will be published separately. We further applied prioritization of FLAGS on an in-house WES/WGS database and illustrated how trio-based exome families have Mendelian recessive and dominant candidates overlapping with the FLAGS. The FLAGS ranking can be fed into the candidate identification process and highlight genes that should be considered as high-risk candidates for false positives [Additional file 14].

## Discussion

WES/WGS studies can identify hundreds to thousands of rare protein-coding mutations per individual. Genes vary in their frequency of appearance; genes that are more likely to harbor rare-coding variants by chance are less likely to be involved in human diseases, especially in the context of rare Mendelian disorders. Previous studies have reported that *TTN* and *MUC16*, the two longest genes in the human genome, should be interpreted with care due to their long lengths [37]-[41]. In this study, we compiled a list of frequently mutated genes (FLAGS) based upon analysis of rare coding mutations from dbSNP and Exome Variant Server ESP6500. We compared the biological properties of FLAGS against genes from disease databases (HGMD, OMIM) that represent the currently best reliable curated resources for disease-associated genes. We further demonstrated the clinical utilities of FLAGS as a gene prioritization tool. The discussion will illustrate additional clinical benefits of FLAGS, and conclude with ideas for future directions and project limitations.

### FLAGS are less likely to be disease-associated

Consistent with our expectations, FLAGS have significantly longer coding lengths, higher average dN/dS ratios, and more paralogs than genes from OMIM and HGMD. Paralogs have been cited as capable to partially compensate for the loss of gene function [42],[43], so the greater frequency of paralogs could mean that mutations are less likely to have a critical impact on phenotype. In the examination of the research literature for FLAGS, we observed fewer disease annotations compared to disease genes, but elevated rates compared to background genes, suggesting that FLAGS have been associated to human disease more frequently than the rest of the protein-coding genes.

### Clinical utilization of FLAGS for prioritization

Prioritizing candidates in rare disease studies is important; as it takes substantial time of experts to review each gene [49], getting better specificity without loss of sensitivity has real value. We demonstrated the utility of FLAGS as a prioritization tool by overlapping FLAGS against candidates from clinical exomes in TIDE, without loss of ultimately identified causal genes. We further illustrated with a single clinical case how when multiple equally attractive candidates are under consideration, FLAGS provide a way for clinicians and researchers to decide which gene to focus on first.

#### Cautionary indicator

While we are not claiming every gene in FLAGS is non-pathogenic, we do wish to make it clear that greater biological evidence is required when interpreting the functional impacts of rare variants in frequently mutated genes. Among the 300 genes with putative pathogenic mutations identified via exome sequencing compiled by Boycott et al. (2013) [7], ten genes intersected with FLAGS. We evaluated the gene-level and variant-level evidence for causality based upon the guideline for investigation of causality published by MacArthur et al. (2014) [23]. We found that many results are derived based upon single-gene sequencing, rather than taking the less biased exome or whole-genome approach [50]-[52]. In addition, many studies reported the mutations as pathogenic simply due to segregation pattern within the family, rare allelic frequency and bioinformatics impact predictions [41],[53]-[55], thus lacking experimental validation at both the variant and gene levels. The screen for rare alleles is further complicated when some of the studies look at minor ethnic populations that are not well represented in the population databases [52],[54],[55]. The evidence behind missense variants is especially doubtful when many missense variants are predicted by CADD [36] to be benign with a lower impact rank than the rare mutations observed from dbSNP and ESP6500. Altogether, these observations could explain why these genes harbor frequent rare functional variations despite being reported in diseases. To avoid false-positive reports of causality, especially for FLAGS, it will be very important for reports to follow the recently published guidelines [56] when assigning pathogenicity to new variants identified as well as additional variants identified in genes previously linked to a particular disease. An example of a good paper would be the one where the variant is identified in a genome-wide screening approach with statistical methods applied to compare the distribution of variants in patients against a large matched control cohorts, where the evidence is assessed at both the candidate gene and candidate variant levels, and where the authors recognize the importance of combining both computational comparative approaches and experimental assays for validating the impact of the variant.

### Going beyond the top 100 and what the future entails

Genes with frequent rare variants need to be appropriately ranked in order to reduce false associations and streamline clinical analysis. Our current results are limited to the top 100 frequently mutated genes. While it may be insightful to study the characteristics of the genes at the other end of the spectrum (the bottom 100 or alternatively sets of genes with low mutation rates and gene-focused publications to exclude genes with poor coverage in exome capture kits), we perceive the greatest long-term utility to be in the incorporation of the complete set of rankings into the exome interpretation process. To make our prioritization ranking accessible to the broad research community, we provide the FLAGS ranking for the genes represented in both dbSNP and EVS.

The novelty that we bring forth is a ranking that utilizes public control exomes/genomes, which clinicians can readily apply to their clinical cases. As discussed above, the ranking is correlated with gene length, evolutionary constraint, and paralogous gene counts.

The high accumulation rate of mutations can be interpreted partially as genes being under less selective constraint. A utility of the FLAGS ranking is that it provides, albeit indirectly, a gene-level indication of the selective constraint upon a gene, while most existing metrics such as phastCons [57] or PhyloP [58] provide a position-specific value. While the FLAGS ranking is not a substitute for the more direct measures, the genic level information complements them.

Current prioritization tools lack the ability to evaluate at both genic and variant level simultaneously. Ultimately, a scoring mechanism integrating biological and technological features at both the genic and variant level should be developed. A future direction is to improve upon methodologies like RVIS [26] and expand beyond the rate of mutation by employing statistical machine learning techniques to incorporate the genic and allelic features as highlighted in this study and previous works to summarize them into a single computational score. Such a new quantitative measurement should improve the ranking of pathogenicity for each gene, and highlight skeptical candidates to accelerate the clinical translation of genomic research findings. The mechanism itself (e.g. the weights of features) would also shed light on the exact nature of the causes of excess mutation rates and facilitate better biological understanding.

In the long-term, the accumulation of more exomes and whole genomes will provide an increasingly rich body of data for the generation of FLAGS rankings.

### Limitations

In the study we relied upon manually-curated GeneRIFs to extract the publications for each gene. One could argue for more sophisticated PubMed queries in combination with semantic rules to increase the sensitivity for assigning human-disease related publications [59],[60]. We also recognize that neither MeSHOP nor HPO capture gene-to-disease terms perfectly. A possible direction is to explore other gene-disease databases such as HuGE Navigator [61]. We further acknowledge that the interpretation of MesHOP and HPO could be influenced by pleiotropic genes. Similarly, we used Ensembl for extracting the paralogous relationships for each gene, but there are other available extraction algorithms and databases for inferring paralogy [62]-[64]. Additionally, our present study is restricted to genes with both an HGNC symbol and a fully annotated translation start and end. We recognize that not all protein-coding genes fit these criteria, and we are excluding non-coding genes (as well as 5′ and 3′ UTRs of coding genes) from this analysis.

## Conclusion

While most complex disorders generally can confirm the strength of their findings by comparing against a matched background cohort, the nature of studying rare monogenic disorders mean that there is often insufficient sample size to conduct a rigorous statistical analysis on the strength of the finding. In this study, we extracted a list of frequently mutated genes based on rare variants from dbSNP and Exome Variant Server. Our results revealed the biological properties of these genes that could explain why they are frequently mutated, and why extra discretion in statistical and biological interpretation needs to be taken when trying to relate these genes to clinical phenotypes. We propose that the ranking of how frequent a gene is mutated in next-generation sequencing studies is useful for the prioritization of candidate genes.

## Consent

Written informed consent was obtained from the patient’s guardian/parent/next of kin for the publication of this report.

## Additional files

## Electronic supplementary material

Additional file 1: Table S1.: This table lists the five datasets used in this study, and the genes that made up each dataset. The first row in the table shows the names of the datasets referred throughout the manuscript, and each column contains the list of genes, referred to by their official gene symbol. (PDF 5 KB)

Additional file 2: Table S6.: A list of variants, in variant call format (VCF), showing the mutations that were observed more than 10 times in our in-house database consisting of 150 exomes and 13 whole genomes, after they were filtered by allelic frequencies according to the annotations from dbSNP and Exome variant server (refer to methodology section for more details). (PDF 9 KB)

Additional file 3: Table S4.: The entire ranked list of FLAGS, with the most frequently mutated genes at the top. (PDF 9 KB)

Additional file 4: Table S2.: There are two lists in this table. The first is a list of genes that overlapped between FLAGS vs. OMIM (Table S2A), and the second is a list of genes that overlapped between FLAGS vs. HGMD (Table S2B). (TXT 220 KB)

Additional file 5: Table S5.: A table showing a comparison of dN/dS ratio between the values we reported with our calculation (see manuscript for methodology), versus a previously published result of a gene set (refer to reference [31] in the manuscript). The results between the two methodologies were highly consistent. (TXT 269 bytes)

Additional file 6: Table S9.: This table shows the two input files that were fed into R for statistical analyses. In the table, the attributes for each gene used in the analysis were described (dN/dS ratio, gene length, # of MeSH terms, # of HPO terms, # of paralogs for table S9A, and # of pathogenic mutations from HGMD for table S9B). (TXT 237 bytes)

Additional file 7: Text S3.: This section contains a concise description of our in-house bioinformatics pipeline for processing exome and whole-genome datasets. (TXT 185 KB)

Additional file 8: Text S4.: This section provides a description of the TIDE-BC project. (TXT 692 bytes)

Additional file 9: Table S7.: A summary of the families studied in TIDEX project, and the number of candidate variants remaining after filtering against genetic and allelic frequency thresholds. The results are broken down by family structure and the types of genetic model applied. Refer to www.tidebc.org and additional text S4 for more information on the TIDEX project, and additional text S3 for how the variants were called and filtered. (ZIP 26 KB)

Additional file 10: Table S8.: A table of number of exomes from TIDEX project that lists out the number of rare functional variants for each protein-coding gene captured in the exome capture kits. Please refer to methodology section for how ‘rare’ and ‘functional’ descriptors were defined. (TXT 2 KB)

Additional file 11: Text S2.: A comparison of our FLAGS gene ranking system against another method, residual variation intolerance score (RVIS) that also built upon public genomic datasets and ranked importance of each gene’s association to human diseases. Agreements and disagreements between the two methods are discussed. (TXT 82 KB)

Additional file 12: Text S5.: This section describes an analysis looking at the distribution of number of MeSH and HPO terms per gene, after normalizing by the number of biological functionally-related literature published for that gene, as reported in GeneRIF. (XLSX 848 KB)

Additional file 13: Text S1.: This section describes an analysis looking at the uniformity of distribution for rare functional variants across genes. The hypothesis was that genes of less significance to monogenic human diseases would display more uniformity in the occurrences of benign coding mutations across the protein sequence, whereas genes that are more linked to causing penetrating diseases would harbor regions that are more devoid of mutations due to conservation of important protein domains. (TXT 161 KB)

Additional file 14: The PDF outlining the supplementary information for this manuscript. (PDF 107 KB)

## Authors’ original submitted files for images

Below are the links to the authors’ original submitted files for images. Authors’ original file for figure 1
Authors’ original file for figure 2
Authors’ original file for figure 3
Authors’ original file for figure 4
Authors’ original file for figure 5
