# Correction to: FLAGS, frequently mutated genes in public exomes

**DOI:** 10.1186/s12920-017-0309-7

**Published:** 2017-11-29

**Authors:** Casper Shyr, Maja Tarailo-Graovac, Michael Gottlieb, Jessica JY Lee, Clara van Karnebeek, Wyeth W Wasserman

**Affiliations:** 1 Centre for Molecular Medicine and Therapeutics, Child and Family Research Institute, Vancouver, BC Canada; 2 Department of Medical Genetics, University of British Columbia, Vancouver, BC Canada; 3 Treatable Intellectual Disability Endeavour in British Columbia, Vancouver, Canada; 4 Bioinformatics Graduate Program, University of British Columbia, Vancouver, BC Canada; 5 Genome Science and Technology Graduate Program, University of British Columbia, Vancouver, BC Canada; 6 Division of Biochemical Diseases, BC Children’s Hospital, Vancouver, BC Canada; 7 Department of Pediatrics, University of British Columbia, Vancouver, BC Canada

## Correction

Unfortunately, the original article [1] contained an error. The additional files were included incorrectly. The correct additional files 1, 2, 3, 4, 5, 6, 7, 8, 9, 10, 11, 12, 13 and 14 are published in this correction.

## Additional files

Additional file 1: Table S1. This table lists the five datasets used in this study, and the genes that made up each dataset. The first row in the table shows the names of the datasets referred throughout the manuscript, and each column contains the list of genes, referred to by their official gene symbol (TXT 220 kb)

Additional file 2: Table S6. A list of variants, in variant call format (VCF), showing the mutations that were observed more than 10 times in our in-house database consisting of 150 exomes and 13 whole genomes, after they were filtered by allelic frequencies according to the annotations from dbSNP and Exome variant server (refer to methodology section for more details). (VCF 207 kb)

Additional file 3: Table S4. The entire ranked list of FLAGS, with the most frequently mutated genes at the top. (TXT 185 kb)

Additional file 4: Table S2A. The list of genes that overlap between OMIM and FLAGS. **Table S2B.** The list of genes that overlap between HGMD and FLAGS. (ZIP 677 bytes)

Additional file 5: Table S5. A table showing a comparison of dN/dS ratio between the values we reported with our calculation (see manuscript for methodology), versus a previously published result of a gene set (refer to reference [31] in the manuscript). The results between the two methodologies were highly consistent. (TXT 692 bytes)

Additional file 6: Table S9A. The list of input attributes for each gene that were fed into R for statistical analyses (dN/dS ratio, gene length, # of MeSH terms, # of HPO terms, and # of paralogs). **Table S9B.** The list of number of pathogenic variants from HGMD for each gene. (ZIP 900 kb)

Additional file 7: Text S3. This section contains a concise description of our in-house bioinformatics pipeline for processing exome and whole genome datasets. (PDF 48 kb)

Additional file 8: Text S4. This section provides a description of the TIDE-BC project. (PDF 49 kb)

Additional file 9: Table S7. A summary of the families studied in TIDEX project, and the number of candidate variants remaining after filtering against genetic and allelic frequency thresholds. The results are broken down by family structure and the types of genetic model applied. Refer to www.tidebc.org and additional text S4 for more information on the TIDEX project, and additional text S3 for how the variants were called and filtered. (TXT 2 kb)

Additional file 10: Table S8. A table of number of exomes from TIDEX project that lists out the number of rare functional variants for each protein-coding gene captured in the exome capture kits. Please refer to methodology section for how ‘rare’ and ‘functional’ descriptors were defined. (TXT 81 kb)

Additional file 11: Text S2. A comparison of our FLAGS gene ranking system against another method, residual variation intolerance score (RVIS) that also built upon public genomic datasets and ranked importance of each gene’s association to human diseases. Agreements and disagreements between the two methods are discussed. (PDF 48 kb)

Additional file 12: Text S5. This section describes an analysis looking at the distribution of number of MeSH and HPO terms per gene, after normalizing by the number of biological functionally-related literature published for that gene, as reported in GeneRIF. (PDF 46 kb)

Additional file 13: Text S1. This section describes an analysis looking at the uniformity of distribution for rare functional variants across genes. The hypothesis was that genes of less significance to monogenic human diseases would display more uniformity in the occurrences of benign coding mutations across the protein sequence, whereas genes that are more linked to causing penetrating diseases would harbor regions that are more devoid of mutations due to conservation of important protein domains. (PDF 47 kb)

Additional file 14: The PDF outlining the supplementary information for this manuscript. (PDF 108 kb)
