# Supplementary figures and images for: FLAGS, frequently mutated genes in public exomes
Source: BMC Med Genomics. 2014 Dec 3;7:64. doi: 10.1186/s12920-014-0064-y (PMC4267152; doi:10.1186/s12920-014-0064-y)

## Distribution of Gene rank

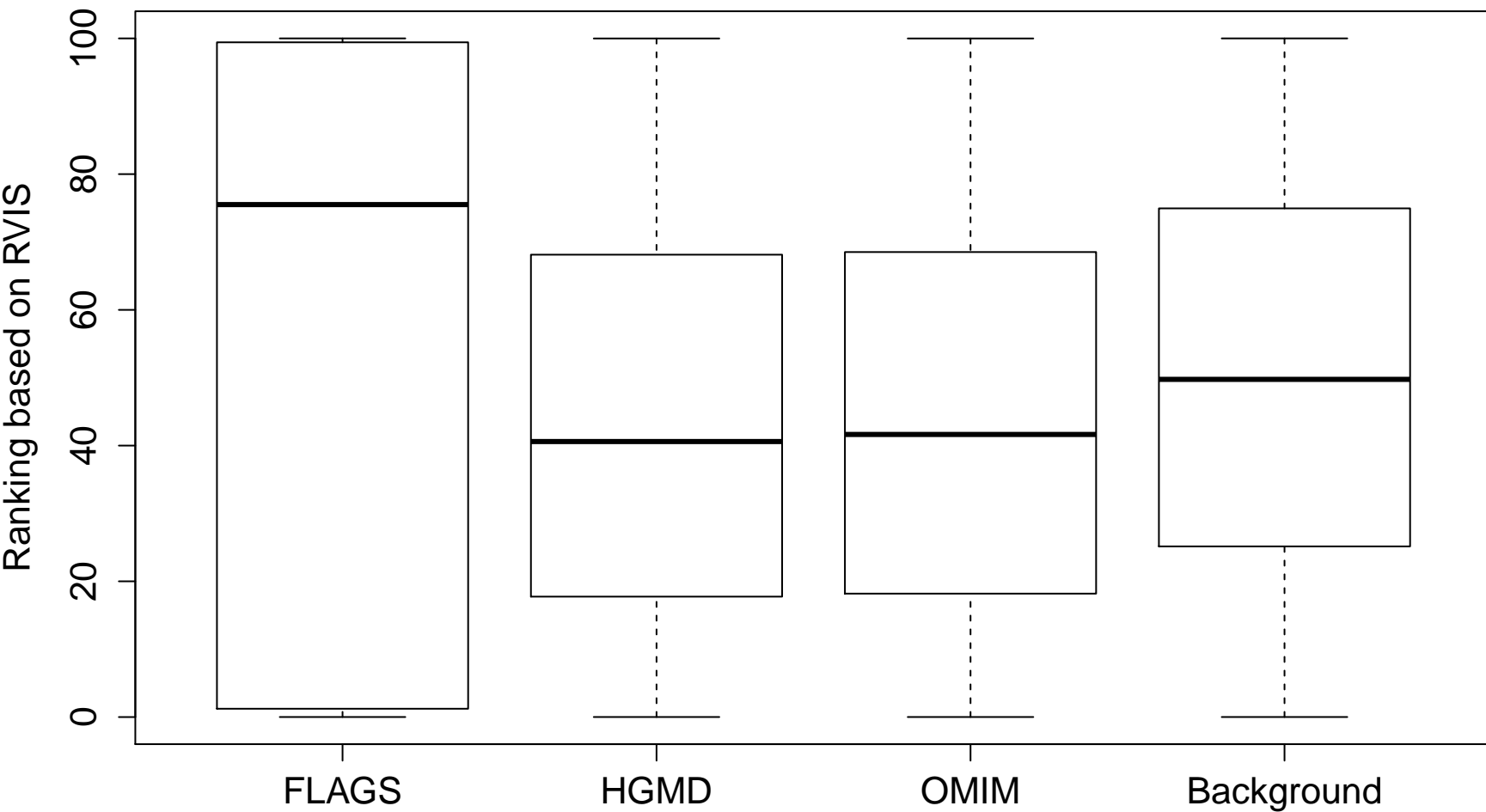

Supplement: Supplementary file 1 — Additional file 1: Table S1.: This table lists the five datasets used in this study, and the genes that made up each dataset. The first row in the table shows the names of the datasets referred throughout the manuscript, and each column contains the list of genes, referred to by their official gene symbol. (PDF 5 KB) [file 12920_2014_64_MOESM1_ESM.pdf]

# Distribution of HPO disease terms

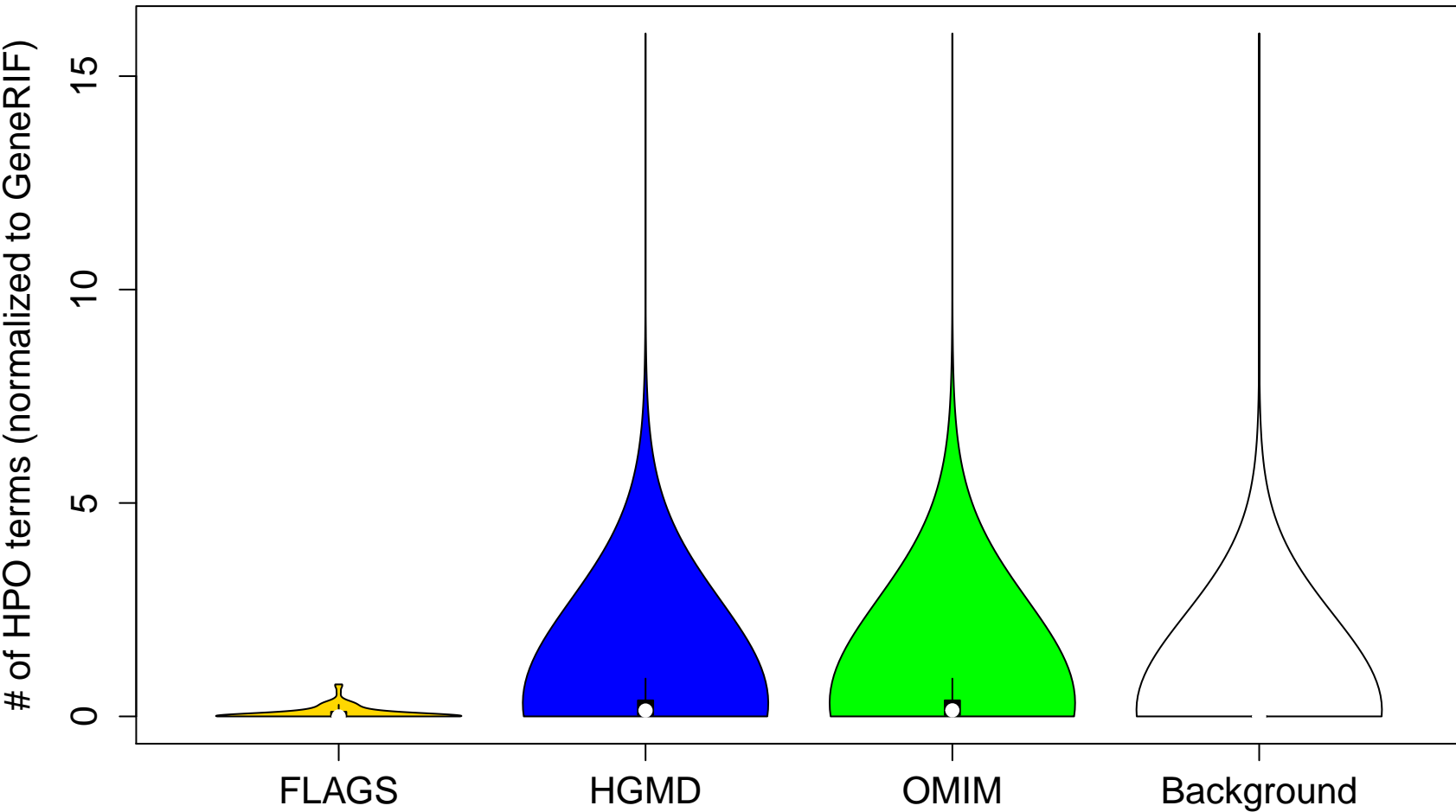

Supplement: Supplementary file 2 — Additional file 2: Table S6.: A list of variants, in variant call format (VCF), showing the mutations that were observed more than 10 times in our in-house database consisting of 150 exomes and 13 whole genomes, after they were filtered by allelic frequencies according to the annotations from dbSNP and Exome variant server (refer to methodology section for more details). (PDF 9 KB) [file 12920_2014_64_MOESM2_ESM.pdf]

# Distribution of MeSH diseased terms

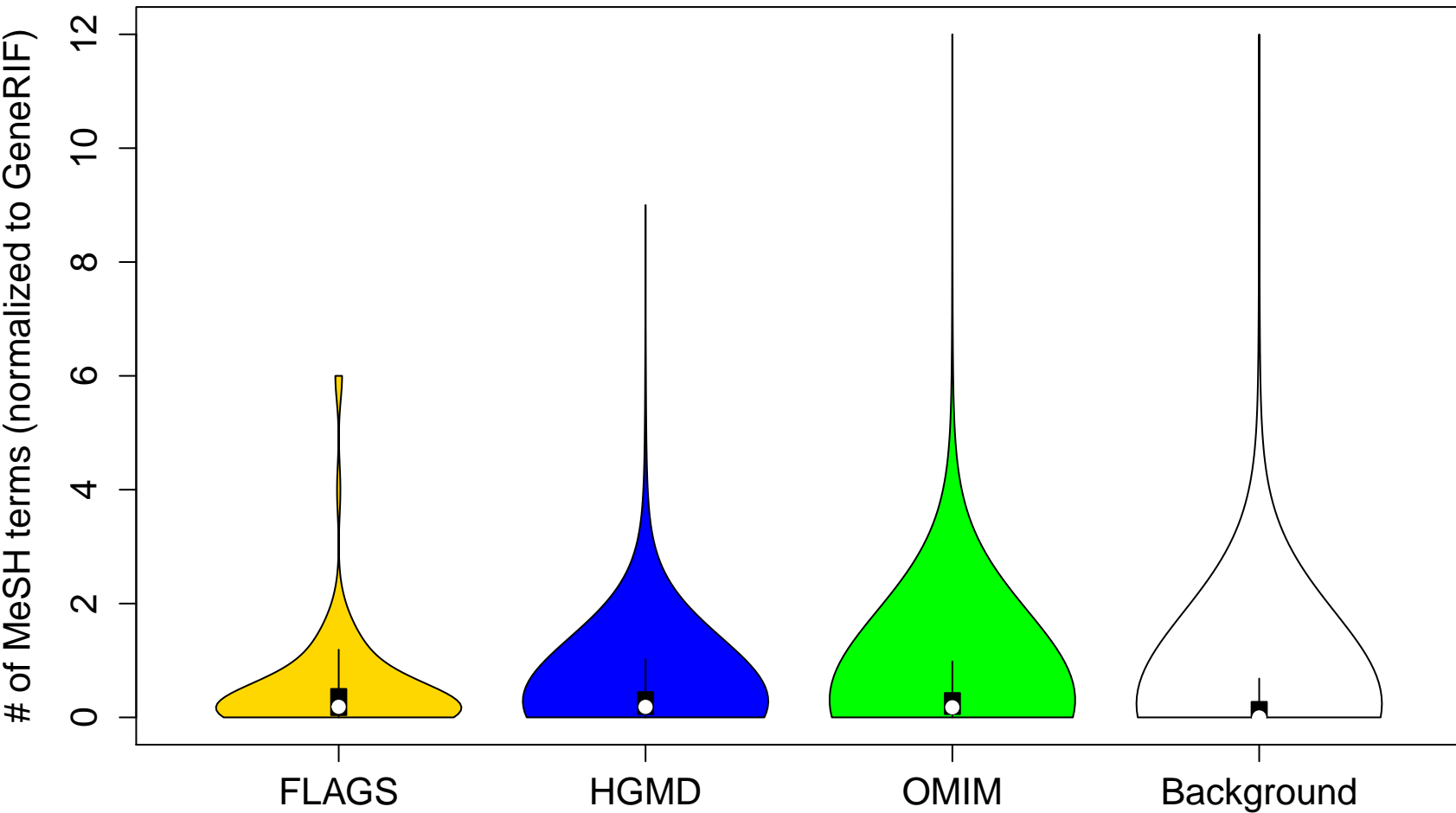

Supplement: Supplementary file 3 — Additional file 3: Table S4.: The entire ranked list of FLAGS, with the most frequently mutated genes at the top. (PDF 9 KB) [file 12920_2014_64_MOESM3_ESM.pdf]

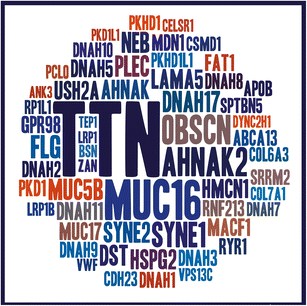

Supplement: Supplementary file 15 — Authors’ original file for figure 1 [file 12920_2014_64_MOESM15_ESM.gif]

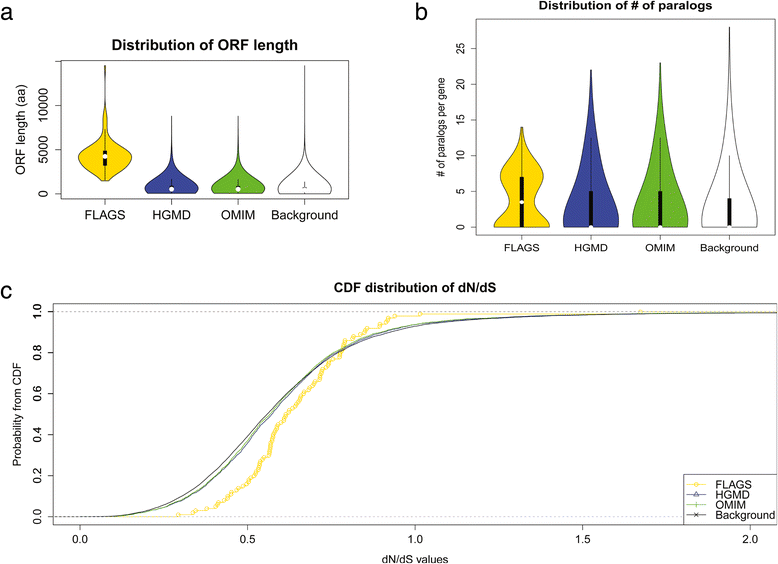

Supplement: Supplementary file 16 — Authors’ original file for figure 2 [file 12920_2014_64_MOESM16_ESM.gif]

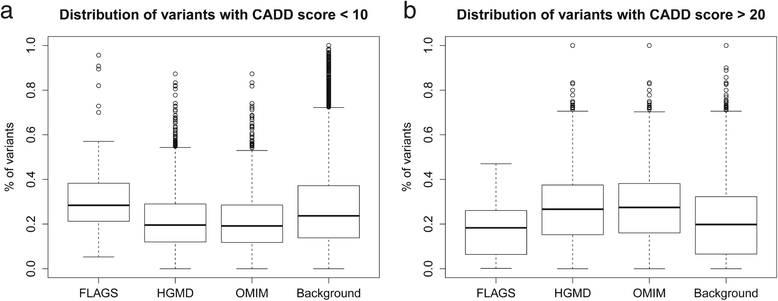

Supplement: Supplementary file 17 — Authors’ original file for figure 3 [file 12920_2014_64_MOESM17_ESM.gif]

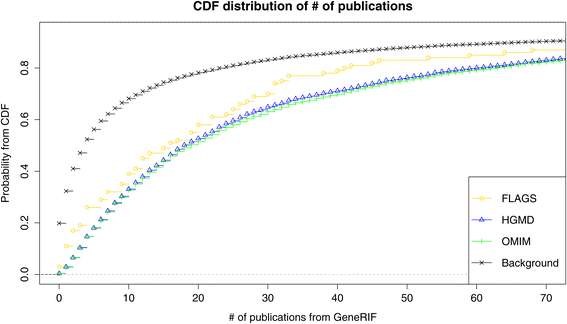

Supplement: Supplementary file 18 — Authors’ original file for figure 4 [file 12920_2014_64_MOESM18_ESM.gif]

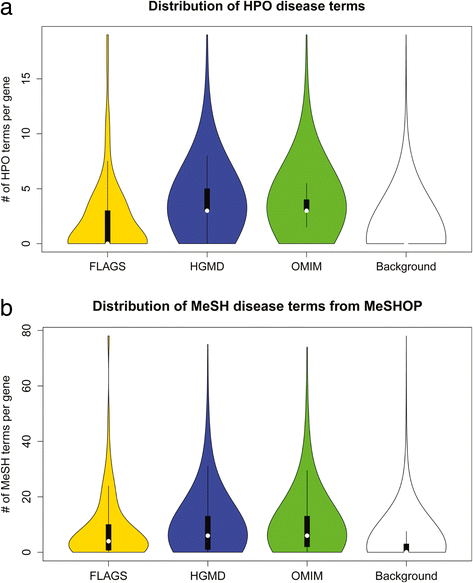

Supplement: Supplementary file 19 — Authors’ original file for figure 5 [file 12920_2014_64_MOESM19_ESM.gif]
