# Supplementary material for: Correction to: FLAGS, frequently mutated genes in public exomes
Source: BMC Med Genomics. 2017 Nov 29;10:69. doi: 10.1186/s12920-017-0309-7 (PMC5706417; doi:10.1186/s12920-017-0309-7)
Supplement: Supplementary file 7 — This section contains a concise description of our in-house bioinformatics pipeline for processing exome and whole genome datasets. (PDF 48 kb) [file 12920_2017_309_MOESM7_ESM.pdf]

S3. In-house bioinformatics pipeline

In this section we discussed briefly the bioinformatics pipeline that we have setup in-house to process clinical exome data from TIDE-BC project. Because the project spans across multiple years, the software and genome versions have undergone various updates, so we will only provide the name of the software used but not the actual version.

The pipeline starts with pair-end 100bp Illumina reads in FASTQ format. The coverage of each exome or whole-genome ranges from as low as 30X to as high as 150X. Reference genome is hg19. Reads are aligned with Bowtie2 aligner under default parameter settings in a cluster server maintained in-house with 13 compute nodes, each with 16 CPUs and 32Gb RAM available per node. Aligned reads are sorted and merged into BAM using Samtools. Reads with < 20 mapping quality score are discarded. Picard adds the read group and library information to the BAM file. GATK performs local re-alignment on the BAM file. BCF file is called from the re-aligned BAM using Samtools. VCF is generated using vcfutils.pl varFilter with mapping quality score 20 and a minimum of 2 alternative bases. Variants from VCF with less than 20 SNP quality score are further filtered out. Variant annotation is done by SNPeff with parameter –SpliceSiteSize 7 using always the latest available genomic annotation available at the time. Custom perl scripts are used to filter variants by Mendelian inheritance models (de novo dominant, homozygous recessive from either one or both parents, compound heterozygous), and filtering against dbSNP database (downloaded from UCSC Genome Browser) and ESP6500 downloaded from Exome variant server, and against the in-house already processed VCFs. Genomic coverage is analyzed using GATK on all the known exons downloaded from Ensembl Biomart. Candidate variants selected for further follow-ups are first manually screened on IGV for quality inspection before Sanger confirmation.
