# Supplementary material for: Correction to: FLAGS, frequently mutated genes in public exomes
Source: BMC Med Genomics. 2017 Nov 29;10:69. doi: 10.1186/s12920-017-0309-7 (PMC5706417; doi:10.1186/s12920-017-0309-7)
Supplement: Supplementary file 8 — This section provides a description of the TIDE-BC project. (PDF 49 kb) [file 12920_2017_309_MOESM8_ESM.pdf]

## S4. TIDE-BC (http://www.tidebc.org)

Tide BC is a new collaborative care & research initiative with a focus on prevention and treatment of Intellectual disability (ID). We have shown that the ID seen in some children is due to treatable genetic conditions known as inborn errors of metabolism (IEM). Many of these IEM's can be treated with diet or drugs. Presently, health care policy and institutional culture is still operating under the old premise that all ID is incurable and thus, many children born with treatable ID are at risk of not being treated. At BC Children's Hospital (BCCH) in Vancouver, Canada, 1500 patients with ID are seen for diagnostic assessment per year by various services, such as neurology, medical genetics, biochemical diseases, developmental pediatrics and child psychiatry. With the local expertise of all these specialists, existing diagnostic laboratory methods, and the major advances in diagnostic and therapeutic technologies, BCCH is the ideal academic location to implement our evidence-based protocol to identify treatable causes of ID. TIDEX was designed by TIDE-BC investigators to take advantage of new technologies to help crack the code for those families who have undergone the million dollar workup and are still unable to receive a diagnosis for their child's debilitating condition. These technological advances, coupled with TIDE-BCs already proven approach, has every promise in providing much needed answers to help those families.

In order to provide those answers, TIDE-BC investigators are presently looking for those undiagnosed patients who have some evidence of an interrupted metabolic pathway or enzyme deficiency. This may be abnormal chemicals in body fluids such as blood or urine or test results that provide a clue that a biochemical pathway may be altered. Then by comparing the protein coding regions or "whole exome" of DNA they hope to find the cause. As sequencing cost continues to decrease, the project now shifts more and more towards whole-genome sequencing, rather than only restricted to exomes. The additional sequencing of one or more healthy family members helps them to eliminate sequence variations that do not contribute to the disorder. The informatics team, based within Dr. Wyeth Wasserman lab, uses a new, CFI-funded computational system. It features high-capacity storage (~0.3 petabytes), a set of high-performance servers supporting virtualized computing, a computing cluster with ~100 computing cores, and a tape system for long-term genome data archiving. The system is interconnected with 10 gigabyte channels for efficiency. Once the genetic cause is found, this group of metabolic disorders are often amenable to simple and successful treatments, sometimes only involving dietary changes or dietary supplementation.
