# Supplementary material for: Correction to: FLAGS, frequently mutated genes in public exomes
Source: BMC Med Genomics. 2017 Nov 29;10:69. doi: 10.1186/s12920-017-0309-7 (PMC5706417; doi:10.1186/s12920-017-0309-7)
Supplement: Supplementary file 11 — A comparison of our FLAGS gene ranking system against another method, residual variation intolerance score (RVIS) that also built upon public genomic datasets and ranked importance of each gene’s association to human diseases. Agreements and disagreements between the two methods are discussed. (PDF 48 kb) [file 12920_2017_309_MOESM11_ESM.pdf]

S2. RVIS

Next, we analyzed the genic tolerance of the FLAGS gene set to variants. We expected FLAGS to be predicted to be more tolerant to variations and thus less likely to be impacted by pathogenic variants resulting in rare human diseases. To investigate this, we used a method published by Petrovski et al. (2013)[3] to assess the residual variation intolerance score (RVIS) for each gene based on their published supplementary dataset. This intolerance scoring system was developed by surveying whether a gene has relatively more or less functional genetic variation compared to the expected value based on neutral variations found in the same gene within the exomes from EVS. We chose this measurement because to our knowledge this is the only reliable published scoring system that is gene-centric rather than variant-centric. For each FLAGS gene, we extracted the relative rank based on the published intolerance score (the lower rank, the more intolerant the gene to variations), and we find that these FLAGS genes have a higher median score of 76 compared to OMIM, HGMD and Background which have medians of 42, 41 and 50 respectively (Supplementary figure 1). However, Mann-Whitney U one-tailed tests revealed no significant differences (p-value between 0.05 and 0.1), likely attributable to the bimodal distribution of the ranks within the FLAGS, as there are genes within the FLAGS that have low RVIS ranks (n=32 with rank < 20). While this supports our findings that majority of the genes in FLAGS are ranked as more tolerant to variations, there are FLAGS that are predicted not to tolerate variation well. We found that these genes tend to have greater proportion of rare functional mutations over polymorphic functional mutations, which may explain why they receive RVIS ranks of <20. Namely, RVIS methodology does not consider rare functional variations, it ranks those genes as intolerant to genetic variation, despite the presence of numerous rare functional variants. We believe this may be a limitation on RVIS, because if a gene is observed to be frequently mutated with rare functional mutations yet is highly ranked as pathogenic in RVIS system, then by expectation that gene should not be highly ranked.

****

Supplementary Figure 1. Distribution of gene ranking across gene sets. The Y-axis plots the boxplot distribution of gene rank based on RVIS score.
****
