# Supplementary material for: Correction to: FLAGS, frequently mutated genes in public exomes
Source: BMC Med Genomics. 2017 Nov 29;10:69. doi: 10.1186/s12920-017-0309-7 (PMC5706417; doi:10.1186/s12920-017-0309-7)
Supplement: Supplementary file 12 — This section describes an analysis looking at the distribution of number of MeSH and HPO terms per gene, after normalizing by the number of biological functionally-related literature published for that gene, as reported in GeneRIF. (PDF 46 kb) [file 12920_2017_309_MOESM12_ESM.pdf]

S5. HPO and MeSH terms normalized to GeneRIF

To adjust for the potential bias that genes with more articles are likely to have more MeSH and HPO terms attached, we repeated the analysis by normalizing the MeSH and HPO terms to the number of publications in GeneRIF. Supplementary figure 2A and 2B show the violin distribution of HPO and MeSH terms per gene after normalization.

****

Supplementary Figure 2A. The Y-axis plots the number of HPO disease terms per gene after normalizing to the number of entries from GeneRIF for the same given gene. FLAGS have significantly fewer terms than OMIM, HGMD and significantly more terms than Background (each p-value << 0.00001; Mann-Whitney 1-tailed test).

****

****

Supplementary Figure 2B. The Y-axis plots the number of MeSH disease terms per gene from MeSHOP after normalizing to the number of entries from GeneRIF for the same given gene. There are no significant differences observed between FLAGS and OMIM and HGMD, but FLAGS have significantly more terms than Background (p-value << 0.00001; Mann-Whitney 1-tailed test)

****
