# Supplementary material for: Correction to: FLAGS, frequently mutated genes in public exomes
Source: BMC Med Genomics. 2017 Nov 29;10:69. doi: 10.1186/s12920-017-0309-7 (PMC5706417; doi:10.1186/s12920-017-0309-7)
Supplement: Supplementary file 13 — This section describes an analysis looking at the uniformity of distribution for rare functional variants across genes. The hypothesis was that genes of less significance to monogenic human diseases would display more uniformity in the occurrences of benign coding mutations across the protein sequence, whereas genes that are more linked to causing penetrating diseases would harbor regions that are more devoid of mutations due to conservation of important protein domains. (PDF 47 kb) [file 12920_2017_309_MOESM13_ESM.pdf]

S1. Distribution of variants across genes

For each gene, only rare coding variants derived from dbSNPv138 and EVS (including short insertions/deletions, i.e. indels) within the longest coding transcript that results in amino acid change were considered. Polymorphic alleles were excluded based on the same allelic frequency criteria as described above. The location of the affected amino acid was derived from annotation by SNPeff[1] software (as described above). For indels, only the first affected amino acid location was considered, such that if an indel affected multiple amino acids, we only considered the location of the first one. To achieve meaningful statistical evaluations, any gene with < 20 remaining variants was not included in this part of the analysis. For each gene with ≥ 20 remaining variants, the same number of variants was randomly selected uniformly across the gene using Python version 2.7 random.randrange function. Mann-Whitney two-sided test was conducted between the locations of the observed mutations versus the locations from randomly selected ones using SciPy's[2] mannwhitneyu function. The p-value from this test was recorded, and the procedure repeated 20,000 times. Treating the p-value as a score, the p-value from this list corresponding to 99% statistical confidence was determined, reflecting how likely is the distribution of the observed variants to deviate from the uniform distribution. A Bonferroni multiple testing correction was applied when interpreting the significance of each p-value.
