# Supplementary material for: Correction to: FLAGS, frequently mutated genes in public exomes
Source: BMC Med Genomics. 2017 Nov 29;10:69. doi: 10.1186/s12920-017-0309-7 (PMC5706417; doi:10.1186/s12920-017-0309-7)
Supplement: Supplementary file 14 — The PDF outlining the supplementary information for this manuscript. (PDF 108 kb) [file 12920_2017_309_MOESM14_ESM.pdf]

SUPPLEMENTARY

Table of Contents

S1. Distribution of variants across genes 2

S2. RVIS 3

S3. In-house bioinformatics pipeline 4

S4. TIDE-BC (http://www.tidebc.org) 5

S5. HPO and MeSH terms normalized to GeneRIF 6

S6. Application in in-house WES /WGS database 7

S1. Distribution of variants across genes

For each gene, only rare coding variants derived from dbSNPv138 and EVS (including short insertions/deletions, i.e. indels) within the longest coding transcript that results in amino acid change were considered. Polymorphic alleles were excluded based on the same allelic frequency criteria as described above. The location of the affected amino acid was derived from annotation by SNPeff[1] software (as described above). For indels, only the first affected amino acid location was considered, such that if an indel affected multiple amino acids, we only considered the location of the first one. To achieve meaningful statistical evaluations, any gene with < 20 remaining variants was not included in this part of the analysis. For each gene with ≥ 20 remaining variants, the same number of variants was randomly selected uniformly across the gene using Python version 2.7 random.randrange function. Mann-Whitney two-sided test was conducted between the locations of the observed mutations versus the locations from randomly selected ones using SciPy's[2] mannwhitneyu function. The p-value from this test was recorded, and the procedure repeated 20,000 times. Treating the p-value as a score, the p-value from this list corresponding to 99% statistical confidence was determined, reflecting how likely is the distribution of the observed variants to deviate from the uniform distribution. A Bonferroni multiple testing correction was applied when interpreting the significance of each p-value.

S2. RVIS

Next, we analyzed the genic tolerance of the FLAGS gene set to variants. We expected FLAGS to be predicted to be more tolerant to variations and thus less likely to be impacted by pathogenic variants resulting in rare human diseases. To investigate this, we used a method published by Petrovski et al. (2013)[3] to assess the residual variation intolerance score (RVIS) for each gene based on their published supplementary dataset. This intolerance scoring system was developed by surveying whether a gene has relatively more or less functional genetic variation compared to the expected value based on neutral variations found in the same gene within the exomes from EVS. We chose this measurement because to our knowledge this is the only reliable published scoring system that is gene-centric rather than variant-centric. For each FLAGS gene, we extracted the relative rank based on the published intolerance score (the lower rank, the more intolerant the gene to variations), and we find that these FLAGS genes have a higher median score of 76 compared to OMIM, HGMD and Background which have medians of 42, 41 and 50 respectively (Supplementary figure 1). However, Mann-Whitney U one-tailed tests revealed no significant differences (p-value between 0.05 and 0.1), likely attributable to the bimodal distribution of the ranks within the FLAGS, as there are genes within the FLAGS that have low RVIS ranks (n=32 with rank < 20). While this supports our findings that majority of the genes in FLAGS are ranked as more tolerant to variations, there are FLAGS that are predicted not to tolerate variation well. We found that these genes tend to have greater proportion of rare functional mutations over polymorphic functional mutations, which may explain why they receive RVIS ranks of <20. Namely, RVIS methodology does not consider rare functional variations, it ranks those genes as intolerant to genetic variation, despite the presence of numerous rare functional variants. We believe this may be a limitation on RVIS, because if a gene is observed to be frequently mutated with rare functional mutations yet is highly ranked as pathogenic in RVIS system, then by expectation that gene should not be highly ranked.

****

Supplementary Figure 1. Distribution of gene ranking across gene sets. The Y-axis plots the boxplot distribution of gene rank based on RVIS score.
****

S3. In-house bioinformatics pipeline

In this section we discussed briefly the bioinformatics pipeline that we have setup in-house to process clinical exome data from TIDE-BC project. Because the project spans across multiple years, the software and genome versions have undergone various updates, so we will only provide the name of the software used but not the actual version.

The pipeline starts with pair-end 100bp Illumina reads in FASTQ format. The coverage of each exome or whole-genome ranges from as low as 30X to as high as 150X. Reference genome is hg19. Reads are aligned with Bowtie2 aligner under default parameter settings in a cluster server maintained in-house with 13 compute nodes, each with 16 CPUs and 32Gb RAM available per node. Aligned reads are sorted and merged into BAM using Samtools. Reads with < 20 mapping quality score are discarded. Picard adds the read group and library information to the BAM file. GATK performs local re-alignment on the BAM file. BCF file is called from the re-aligned BAM using Samtools. VCF is generated using vcfutils.pl varFilter with mapping quality score 20 and a minimum of 2 alternative bases. Variants from VCF with less than 20 SNP quality score are further filtered out. Variant annotation is done by SNPeff with parameter –SpliceSiteSize 7 using always the latest available genomic annotation available at the time. Custom perl scripts are used to filter variants by Mendelian inheritance models (de novo dominant, homozygous recessive from either one or both parents, compound heterozygous), and filtering against dbSNP database (downloaded from UCSC Genome Browser) and ESP6500 downloaded from Exome variant server, and against the in-house already processed VCFs. Genomic coverage is analyzed using GATK on all the known exons downloaded from Ensembl Biomart. Candidate variants selected for further follow-ups are first manually screened on IGV for quality inspection before Sanger confirmation.

S4. TIDE-BC (http://www.tidebc.org)

Tide BC is a new collaborative care & research initiative with a focus on prevention and treatment of Intellectual disability (ID). We have shown that the ID seen in some children is due to treatable genetic conditions known as inborn errors of metabolism (IEM). Many of these IEM's can be treated with diet or drugs. Presently, health care policy and institutional culture is still operating under the old premise that all ID is incurable and thus, many children born with treatable ID are at risk of not being treated. At BC Children's Hospital (BCCH) in Vancouver, Canada, 1500 patients with ID are seen for diagnostic assessment per year by various services, such as neurology, medical genetics, biochemical diseases, developmental pediatrics and child psychiatry. With the local expertise of all these specialists, existing diagnostic laboratory methods, and the major advances in diagnostic and therapeutic technologies, BCCH is the ideal academic location to implement our evidence-based protocol to identify treatable causes of ID. TIDEX was designed by TIDE-BC investigators to take advantage of new technologies to help crack the code for those families who have undergone the million dollar workup and are still unable to receive a diagnosis for their child's debilitating condition. These technological advances, coupled with TIDE-BCs already proven approach, has every promise in providing much needed answers to help those families.

In order to provide those answers, TIDE-BC investigators are presently looking for those undiagnosed patients who have some evidence of an interrupted metabolic pathway or enzyme deficiency. This may be abnormal chemicals in body fluids such as blood or urine or test results that provide a clue that a biochemical pathway may be altered. Then by comparing the protein coding regions or "whole exome" of DNA they hope to find the cause. As sequencing cost continues to decrease, the project now shifts more and more towards whole-genome sequencing, rather than only restricted to exomes. The additional sequencing of one or more healthy family members helps them to eliminate sequence variations that do not contribute to the disorder. The informatics team, based within Dr. Wyeth Wasserman lab, uses a new, CFI-funded computational system. It features high-capacity storage (~0.3 petabytes), a set of high-performance servers supporting virtualized computing, a computing cluster with ~100 computing cores, and a tape system for long-term genome data archiving. The system is interconnected with 10 gigabyte channels for efficiency. Once the genetic cause is found, this group of metabolic disorders are often amenable to simple and successful treatments, sometimes only involving dietary changes or dietary supplementation.

S5. HPO and MeSH terms normalized to GeneRIF

To adjust for the potential bias that genes with more articles are likely to have more MeSH and HPO terms attached, we repeated the analysis by normalizing the MeSH and HPO terms to the number of publications in GeneRIF. Supplementary figure 2A and 2B show the violin distribution of HPO and MeSH terms per gene after normalization.

****

Supplementary Figure 2A. The Y-axis plots the number of HPO disease terms per gene after normalizing to the number of entries from GeneRIF for the same given gene. FLAGS have significantly fewer terms than OMIM, HGMD and significantly more terms than Background (each p-value << 0.00001; Mann-Whitney 1-tailed test).

****

****

Supplementary Figure 2B. The Y-axis plots the number of MeSH disease terms per gene from MeSHOP after normalizing to the number of entries from GeneRIF for the same given gene. There are no significant differences observed between FLAGS and OMIM and HGMD, but FLAGS have significantly more terms than Background (p-value << 0.00001; Mann-Whitney 1-tailed test)

****

S6. Application in in-house WES /WGS database

To further demonstrate the utility of this study, we evaluated how frequently FLAGS appear as gene candidates in an in-house collection of 150 exomes and 13 whole genomes – comprising of 53 independent families suffering from distinct rare inborn errors of metabolism (IEM) (http://www.tidebc.org). These cases represent a collection of exome and whole genomes collected over a period of 3 years to study rare intellectual disorders exhibiting metabolic defects. Each family displayed a unique undiagnosed IEM, and the family structures range from singleton case (i.e. proband only) to paired (mother-proband; proband-affected sibling) to trio (father-mother-proband) to quartet (father-mother-proband-sibling) [for more details on exact breakdown of family structure, see Supplemental table 7]. In each family, rare functional variants falling into Mendelian inheritance patterns were extracted by an in-house pipeline (Supplemental text S3), which we then overlapped against FLAGS. When focusing only on the top 100 frequently mutated genes from FLAGS, on average across all 53 families, we see ~3 genes from the recessive models overlapping with the FLAGS per family, which is around ~8% of the recessive candidates per family. From the de novo dominant model, on average ~4 genes overlapped with FLAGS, which is around ~3% of the de novo candidates per family. This demonstrates that many top genes in FLAGS do indeed show up at a relatively frequent rate across exome families despite after applying rigorous canonical filtering at the variant level. While these results are drawn from data processed by an in-house pipeline based on a specific class of disorder, our processing methodology is built on popular tools setup in a workflow as recommended by Broad Institute (http://www.broadinstitute.org/gatk/guide/best-practices) using standard parameters and common filtering strategies[1] such that they should be reproducible in other labs using a similar approach in studying other classes of rare Mendelian disorders.

1. Gilissen C, Hoischen A, Brunner HG, Veltman JA: **Unlocking Mendelian disease using exome sequencing**. *Genome Biol* 2011, **12**:228.
